# From historical to interoperable data: Darwin Core Standardisation of selected carabid beetles records in Italy (1977–1993)

**DOI:** 10.3897/BDJ.14.e186831

**Published:** 2026-07-02

**Authors:** Fabiola Durante, Roberto Pizzolotto

**Affiliations:** 1 Università della Calabria, Rende (CS), Italy Università della Calabria Rende (CS) Italy

**Keywords:** Carabidae, Coleoptera, Darwin Core, GBIF, soil fauna

## Abstract

**Background:**

Carabid beetles (Coleoptera) are a group of living organisms with characteristics that make them suitable as model organisms for ecological studies, because they are good bioindicators and one of the most widespread beetle families in the world. Although numerous scientific works are available on the different aspects of carabid biology, there is a lack of open and standardised data. This work focuses on carabids present in various Italian habitats, from forests to pastures. Their presence was recorded across mountain areas of Italy, specifically in the Apennines (Tuscan-Emilian, Abruzzese, Lucanian, Calabrian and Sicilian Apennines) and in the Alpine area of the Lessini mountains, in natural and semi-natural environments, located at altitudes ranging from 150 to 1840 m a.s.l. The aim of this work is to develop a dataset from a selected pool of studies, standardise it according to the Darwin Core standard and make it freely accessible to the scientific community. This approach facilitates data integration, sharing and reuse, promoting transparency and collaboration in ecological research.

**New information:**

This study provides a unified and standardised dataset combining previously dispersed sources, some of which were in Italian and not easily accessible. This new dataset adheres to the FAIR data principles (Findable, Accessible, Interoperable, Reusable) and contributes to feed the information on carabid beetles already stored in the Global Biodiversity Information Facility (GBIF). The dataset incorporates new elements not included in the original publications: activity densities were calculated for species represented by a single individual in the research of De Felici and collaborators (1995) and missing site information from the research of Brandmayr and Pizzolotto (1990) was completed with four additional sites. By harmonising these records according to Darwin Core standards, the dataset enhances data integration and reuse, demonstrating the value of standardisation and "FAIRification" for biodiversity research and enabling the scientific community to access a comprehensive, high-quality resource on Italian carabid beetles communities.

## Introduction

The digitalisation of legacy scientific literature provides an opportunity to mobilising and mining historical biodiversity data (Nelson and Ellis 2018, Hedrick et al. 2020), either as species lists (detailing species-region relationships) or biocenotic assemblages (detailing species-ecosystem relationships). However, these papers cannot always be directly searched or mined by computers. This is often due to "image-only" PDF formats or a lack of Digital Object Identifiers (DOIs). Even when data can be extracted from properly encoded PDFs, the information must still be "interpreted" according to the specific methodologies outlined in each paper (Ogilvie 2016).

In this context, the curation of old literature is fundamental for retrieving information on flora and fauna. This enhances our understanding of historical trends and enables comparisons between past and present conditions, a task that is especially critical in light of current global climate change (Farrell et al. 2022).

Historical research on Italian ground beetles (Carabidae) has not always been published in digital formats. Consequently, many studies remain available only in hardcopy, often preserved in personal archives. This lack of digitisation makes them difficult to locate, effectively restricting access to data that could be fundamental to broader scientific research.

This is precisely the case for the datasets discussed here; several past studies do not align with FAIR principles. As a result, they are not openly available to the international scientific community and can only be accessed by making a personal request to the original authors. Due to this lack of findability regarding originally hardcopy-published papers, the FAIRification of data is currently limited by the specific knowledge or physical materials available to the paper curator.

The aim of this paper is to describe a dataset, based on research conducted between 1977 and 1993, focusing on carabid beetle communities inhabiting various Italian forests and pastures across the Apennines and the Alps.

### Object of study

The main objective of this study was to curate scientific literature, both in digital and paper format, to extract data on carabid beetles and subject them to a standardisation process through the Darwin Core standard. This approach promotes the information contained in selected works, since their publication in this new format makes them suitable for the FAIR principles, thus, in line with the increasing demand for FAIR data, in order to improve scientific activities (Wilkinson et al. 2016). It is, therefore, believed that data published in open format, through the Darwin Core standard and in freely accessible databases, take on new value and can further support collaboration between researchers within the scientific community.

A further aim was to make available an open dataset contributing to the study of the Italian woodland environments through the evaluation of a specific taxon of ground-dwelling beetles. Specifically, the investigated environments are mostly represented by beech forests (*Fagus sylvatica* L.) with 21 of the 45 reported sites (46.7%), a tree species widely distributed in Europe, with great historical economic importance for timber production (Mazza et al. 2023), followed by pastures with eight sites (17.8%) and oak forests with five sites (11.1%).

### Source identification and inclusion criteria

The identification of sources did not follow a systematic protocol. A targeted, non-systematic approach was adopted, based on the reading and analysis of documents of help to feed the initiative started in 2021 (Pizzolotto 2022) to make available an open dataset on Italian carabid beetles. This strategy was considered appropriate given the nature of the study, which aimed to recover, standardise and harmonise biodiversity data scattered across heterogeneous, partially inaccessible and historically fragmented sources.

Thus, in general, the selection principle was to seek documentation that characterised carabid beetle communities in different habitats, from woodland environments, especially beech or oak forests, to non-forest environments, such as pastures.

The identified publications are scientifically relevant, but some of them lack a Digital Object Identifier (DOI), as they were published before the widespread adoption of DOI registration. Lack of DOI is a feature of historical literature, due to publication date, but also to editorial context. However, where available, DOI or web url were recorded to ensure traceability and interoperability of the associated data, after identification through accessible electronic repositories and journal archives.

Potential selection bias is acknowledged. However, the primary objective of this work was data recovery, standardisation and integration of historically fragmented biodiversity information, which justifies the adoption of a targeted, non-systematic approach. Although non-systematic, this methodology enabled the construction of a coherent, traceable and thematically focused dataset derived from historical, semi-accessible and sometimes digitally available sources.

### Data limitations and reuse guidelines

The dataset integrates carabid assemblage data derived from multiple studies conducted across different geographic regions (Alpine, Apennines and Mediterranean systems) and time periods. Differences amongst studies include variation in sampling effort, trap density, seasonal coverage and duration of pitfall trap exposure. Such heterogeneity may influence observed activity density, species richness and community structure. Consequently, quantitative comparisons of abundance across studies should be interpreted cautiously, possibly after data transformation (as, for example, in Patil et al. (2022)). The dataset is more suitable for analyses, based on species occurrence, compositional patterns and ecological structure rather than direct comparisons of absolute abundance or long-term population trend.

Overview of selected articles:

1. Brandmayr and Zetto Brandmayr (1986): ecological-faunistic study aimed at analysing the communities of Carabid and Rhysodid beetles in beech and fir forests of the Apennines. The research, conducted in 1976-1977, involved 14 mountain forest sites distributed along the Apennines range. Sampling was carried out through direct collection and pitfall traps. Around 20,000 specimens belonging to approximately 60 species were collected;
2. Pizzolotto and Brandmayr (1990): this ecological study on carabid beetle coenoses was conducted between 1982 and 1983 in the Nebrodi Mountains (Province of Messina, Sicily). The study area covered various biotopes from evergreen cork oak forests at lower altitudes to beech and Turkey oak forests in the higher elevations (above 1300 m), also including pastures and shrublands. Data for sites not previously reported in literature (Su, CS, Ce and Fa, see Table 1 and section Additional information in Data resources for details) were retrieved from samples not yet identified, preserved in archives of the Dept. B.E.S.T of the Calabria University;
3. Chemini and Pizzolotto (1992): this research, conducted in 1982, is a community ecology study, focused on the biodiversity and distribution of carabid beetles in forest environments. The study took place in the Lessini Mountains (Province of Trento), across five representative woodland sites, specifically focusing on two types of forests the Ostrya woods (*Ostrya carpinifolia*) and the beech (*Fagus sylvatica*) forests. Sampling was conducted by using pitfall traps and 22 were the species collected;
4. Pizzolotto (1993): the research was conducted in 1987 across several forest stations on the Aspromonte Mount, which mainly included beech and fir forests, with variations in altitude, exposure and slope of the terrain. Sampling was carried out using pitfall traps and direct collection. More than 8,000 specimens belonging to 43 species have been collected. The main objective, in addition to the description and mapping of communities of carabid beetles in relation to major vegetation types, was the production of a concise assessment, based on species traits in order to obtain an Environmental Impact Assessment (EIA).
5. De Felici et al. (1995): the study was conducted at the ENEA Center in Brasimone (Apennines) between 1992 and 1993, across various forest and meadow habitats. Using pitfall traps, 13,607 individuals belonging to 62 species were collected. Table 2 in De Felici et al. (1995) lists the accidental species for which the activity densities were calculated in the present paper, using the formula reported in the "Sampling methods" section. The ultimate goal of the research was to evaluate the communities of carabids in forest and meadow formations with different features, structures and management. The sites involved were high-stem forests, coppice forests with different forest management, but similar ecological characteristics, plus a sample site in an uncultivated area.

The curation of historical literature often finds limitations inherent in the nature of the curated papers, as for the proposed dataset, where the lack of detailed information for each individual trap may lead to reduced accuracy in the estimates. However, the dataset does include general information on the number of active traps, their structure, their contents, the exposure period expressed in days or, in some cases, their arrangement along the transect expressed in metres.

## General description

### Purpose

The aim of this work is to collect in a single dataset a selected number of field researches on carabid beetles conducted in various Italian environments, mainly forests, such as beech or oak forests, but also non-forest environments, such as pastures from Alps to Apennines.

### Additional information

At the event level, site names were standardised within the dwc:eventID field by adopting a consistent format that includes the sampling year, the geographical reference and the specific site name as reported in the original tables (e.g. year_georeference_sitename), while the dwc:habitat field was standardised using EUNIS codes to ensure a uniform classification across all sites. Similarly, individual occurrences were documented in the dwc:occurrenceID field using a derived format that maintains a direct link to the sampling event. This identifier was structured by concatenating the eventID with the first four letters of the genus and the first four letters of the specific epithet of the species under examination (e.g. eventID_genus4_specificepithet4). This approach ensures a hierarchical and traceable connection between each taxonomic record and its respective sampling context.

Other environmental parameters, such as aspect, slope, inclination or vegetation cover were not standardised; instead, these variables were aggregated and reported in Table 1, exactly as described in the original source articles.

Another innovative element resulting from this work is the publication of new information that was added to the original data, thus making the datasets complete and available for the first time.

## Sampling methods

### Sampling description

Carabid beetles were collected between 1977 and 1993, sampling different locations in different years by pitfall traps. At the end of every sampling campaign, a “year’s sample” was obtained, which is the sum of the collections made during the research campaign.

The sampling protocols were similar to the research of Brandmayr and Zetto Brandmayr (1986), Brandmayr and Pizzolotto (1990), Pizzolotto (1993) and De Felici et al. (1995), where pitfall traps were plastic cups, buried up to the edge, 11 cm depth, with a diameter at the mouth of 9.2 cm. A hole at 2/3 from the bottom was made to facilitate the outflow of rainwater. Inside they contained 200 ml of a mixture of vinegar with the addition of 5% formalin. The samples from Chemini and Pizzolotto (1992) were gathered by means of pitfall traps with a mouth diameter of 7 cm and a depth of 9 cm, with a saturated solution of potassium bichromate (K_2_Cr_2_O_7_).

The species abundance was evaluated as annual Activity Density (aAD). Given the unpredictable events that cause traps to break (e.g. cows, tourists), the number of individuals sampled in a year within each site (year sample) may be affected by an uneven sampling effort. For this reason, aAD was evaluated on the total annual number of individuals caught and on the total annual sampling effort unit (EU), related to a period of ten days, as follows:

eu = (traps * days) / 10;

eu is the effort unit for each single sampling period from which the total annual capture effort (EU) is given by the sum of:

EU = ∑eu

and, therefore,

aAD = total number of individuals/EU.

## Geographic coverage

### Description

In Table 1, the main features of the sampled sites were given. Detailed description of the sample sites in the referenced literature. The reported altitude corresponds to the average specific traps placement location, for slopes, the difference in values (single or intervals) is due to the different way of representing the data in the different works. Marked with the symbol (*) are the four new sites published for the first time. Habitat representative code refers to "EUNIS classification".

### Coordinates

37.908 and 45.745 Latitude; 11.053 and 15.956 Longitude.

## Taxonomic coverage

### Taxa included

**Table:** 

Rank	Scientific Name	Common Name
family	Carabidae	Carabids

## Temporal coverage

**Data range:** 1977-5-24 – 1993-1-12.

## Usage licence

### Usage licence

Other

### IP rights notes

CC BY-NC 4.0

## Data resources

### Data package title

Carabid beetles in Italian forests: a dataset from curated literature

### Resource link

https://doi.org/10.15468/92a3j5

### Number of data sets

2

### Data set 1.

#### Data set name

Event

#### Data format

Darwin Core table (tab delimited).

#### Description

Table of the sampled sites. 45 sites (records) were sampled within 150–1840 m a.s.l. altitude, including pastures, beech forests and oak forests. See text for details.

**Data set 1.** 

Column label	Column description
eventID	An identifier for the set of information associated with a dwc:Event (something that occurs at a place and time). Here, an identifier specific for each sample site was generated by composing the sampling year with the locality and the original code given in the publications.
samplingProtocol	The names of, references to, or descriptions of the methods or protocols used during a dwc:Event.
sampleSizeValue	Number of active pitfall traps.
sampleSizeUnit	One pitfall trap.
samplingEffort	∑traps*(days/10).
eventDate	The date-time or interval during which a dwc:Event occurred.
year	The four-digit year in which the dwc:Event occurred, according to the Common Era Calendar.
startDayOfYear	The earliest integer day of the year on which the dwc:Event occurred.
endDayOfYear	The latest integer day of the year on which the dwc:Event occurred.
countryCode	The standard code for the country in which the dcterms:Location occurs.
verbatimElevation	The original description of the elevation (altitude, usually above sea level) of the Location.
habitat	A category or description of the habitat in which the dwc:Event occurred. The EUNIS habitat classification was applied, followed by the link to the description.
decimalLatitude	Latitude in decimal degrees.
decimalLongitude	Longitude in decimal degrees.
geodeticDatum	The ellipsoid, geodetic datum or spatial reference system (SRS), upon which the geographic coordinates given in dwc:decimalLatitude and dwc:decimalLongitude are based.
coordinateUncertaintyInMetres	The horizontal distance (in metres) from the given decimalLatitude and decimalLongitude describing the smallest circle containing the whole of the Location.
locality	Geographic information of the place where sample sites were located.
fieldNotes	The notes taken in the field about the dwc:Event.
eventRemarks	Comments or notes about the dwc:Event.
country	The name of the country in which the dcterms:Locality occurs.
minimumElevationInMetres	The lower limit of the range of elevation (altitude, usually above sea level), in metres.
maximumElevationInMetres	The upper limit of the range of elevation (altitude, usually above sea level), in metres.

### Data set 2.

#### Data set name

Occurrence

#### Data format

Darwin Core table (tab delimited).

#### Description

803 records (i.e. sample sites, see previous table) where the annual Activity Density (AD) of 177 species was given.

**Data set 2.** 

Column label	Column description
eventID	An identifier for the set of information associated with a dwc:Event (something that occurs at a place and time). Here, an identifier specific for each sample site (the same as for the event table).
occurrenceID	An identifier for the Occurrence, possibly a persistent global unique identifier. Here, it is given by the eventID plus the four first letters of genus and species.
basisOfRecord	The specific nature of the data record. Here, all records come from human observation.
associatedReferences	A list (concatenated and separated) of identifiers (publication, bibliographic reference, global unique identifier, URI) of literature associated with the dwc:Occurrence.
recordedBy	A list (concatenated and separated) of names of people, groups or organisations responsible for recording the original dwc:Occurrence.
individualCount	The number of individuals present at the time of the dwc:Occurrence.
organismQuantity	A number or enumeration value for the quantity of organisms. Here, an index of activity density (see organismQuantityType column).
organismQuantityType	The type of quantification system used for the quantity of organisms. Here, the activity density = individualCount/sampling effort.
lifeStage	The age class or life stage of the dwc:Organism(s) at the time the dwc:Occurrence was recorded.
occurrenceStatus	A statement about the presence or absence of a dwc:Taxon at a Location.
scientificName	The full scientific name.
kingdom	The full scientific name of the kingdom in which the species is classified.
phylum	The full scientific name of the phylum in which the species is classified.
class	The full scientific name of the class in which the species is classified.
order	The full scientific name of the order in which the species is classified.
family	The full scientific name of the family in which the species is classified.
genus	The full scientific name of the genus in which the species is classified.
specificEpithet	The name of the species epithet of the dwc:scientificName.
infraspecificEpithet	The name of the lowest or terminal infraspecific epithet of the dwc:scientificName, excluding any rank designation.
taxonRank	The taxonomic rank of the most specific name in the dwc:scientificName.

## Additional information

The most common species are highlighted in Table 2. The Frequency is the percentage of presences in the 45 sampled sites. The Total aAD (activity density) is the sum of each species' annual Activity Density (aAD) in all sampled sites.

In this paper, it was decided to complete two works, the authors of which decided to exclude certain information from the original publications. Specifically, these are the works of Brandmayr and Pizzolotto (1990) and De Felici et al. (1995). In the first case, the authors decided to omit four sites from the list of species, because the collected specimens were still not identified at the time of publication. Some information on this work has also been taken from another publication (Pizzolotto and Brandmayr 1990). In the second case, the authors decided to not evaluate the activity density of 18 species present in the study areas with single individuals, defined as accidental species. On the basis of the sampling effort given by the authors, it was possible to compute the activity density of those 18 species and to include them in the final dataset, contributing in this way to a completion of the primary data.

## Figures and Tables

**Table 1.** Main features of the sample sites. Sample site identificator was generated by composing the sampling year with the locality and the original code given in the publications. The slope in the Lessini and ToscoEm sites was in percentage, here converted in degrees from the original. Habitat according to EUNIS classification.

Sample site	Altitude (metres a.s.l.)	Aspect	Slope	Active Traps (n.)	Veg. cover	Habitat
1984_Term1FagIBP	1680 m	SE	15-20°	6	88%	EUNIS: T1751 Alpino-Apennine neutrophile Fagus forests
1984_Term2CScam	1645 m	NNE	28°	8	100%	EUNIS: T1751 Alpino-Apennine neutrophile Fagus forests
1984_Maje2GSLeon	1450 m	OSO	10-15°	8	90%	EUNIS: T1751 Alpino-Apennine neutrophile Fagus forests
1984_Maje1GSLeon	1440 m	ONO	30°	8	83%	EUNIS: T1751 Alpino-Apennine neutrophile Fagus forests
1984_Maje3PLanc	1400 m	ENE	17-19°	9	95%	EUNIS: T1751 Alpino-Apennine neutrophile Fagus forests
1984_Pollino1ColImp	1580 m	NO	15°	10	90%	EUNIS: T1763 Pollino Fagus forests
1984_Pollino2PZapern	1480 m	S-SSO	15-20°	6	100%	EUNIS: T1763 Pollino Fagus forests
1982_Lessini_A	150 m	W	17°	10	90-100%	EUNIS: T19B13 Montane hop-hornbeam forests
1982_Lessini_B	675 m	E	0-6°	10	70-80%	EUNIS: T19B13 Montane hop-hornbeam forests
1982_Lessini_C	1220 m	W	22°	10	80-90%	EUNIS: T17 Fagus forest on non-acid soils
1982_Lessini_D	1300 m	W	6-22°	10	80-90%	EUNIS: T17 Fagus forest on non-acid soils
1982_Lessini_E	1410 m	W	24-31°	10	90-100%	EUNIS: T17 Fagus forest on non-acid soils
1987_Asprom_P	1360 m	S	20°	10	65%	EUNIS: R1R Mediterranean to Atlantic open, dry, acid and neutral grassland
1987_Asprom_PI	1390 m	E	0-15°	6	65%	EUNIS: T375 Calabrian Pinus laricio forests
1987_Asprom_Q	1380 m	E	20°	6	40%	EUNIS: T1951 Southern Italic subthermophilous oak forests
1987_Asprom_AF1	1360 m	SW	15°	6	95%	EUNIS: T1765 Aspromonte Fagus forests
1987_Asprom_AbF	1630 m	E	30°	6	90%	EUNIS: T331 Southern Apennine Abies alba forests
1987_Asprom_AF2	1280 m	N	25°	5	100%	EUNIS: T1765 Aspromonte Fagus forests
1987_Asprom_RM	1510 m	E	0°	5	10-80%	EUNIS: T1765 Aspromonte Fagus forests
1987_Asprom_AF3	1300 m	N	30°	6	95%	EUNIS: T1765 Aspromonte Fagus forests
1987_Asprom_Ag	1330 m	E	5°	6	80%	EUNIS: T121 Riverine Fraxinus - Alnus forest, wet at high, but not at low water
1990_Nebrodi_S2	1460 m	N-NE	20°	9	95%	EUNIS: T1766 Northern Sicilian Fagus forests
1990_Nebrodi_S2a	1475 m	SSW	10°	5	80%	EUNIS: T1766 Northern Sicilian Fagus forests
1990_Nebrodi_M1	1215 m	WNW	20°	9	75%	EUNIS: T1766 Northern Sicilian Fagus forests
1990_Nebrodi_M1a	1300 m	W	20°	4	95%	EUNIS: T1766 Northern Sicilian Fagus forests
1990_Nebrodi_S5	1290 m	NE	15°	9	85%	EUNIS: T19511 Southern Italic Quercus cerris forests
1990_Nebrodi_M2	1185 m	W	18°	10	85%	EUNIS: T19511 Southern Italic Quercus cerris forests
1990_Nebrodi_M3	1200 m	WNW	5°	6	90%	EUNIS: S51J5 Calicotome brush
1990_Nebrodi_M3a	1200 m	WNW	5°	4	50%	EUNIS: S51J5 Calicotome brush
1990_Nebrodi_M4a	1335 m	ENE	12°	4	80%	EUNIS: R21 Mesic permanent pasture of lowlands and mountains
1990_Nebrodi_S1	1840 m	ENE-E	10°	6	40%	EUNIS: R14 Perennial rocky grassland of the Italian Peninsula
1990_Nebrodi_S1a	1840 m	NE	10°	3	50%	EUNIS: R14 Perennial rocky grassland of the Italian Peninsula
1990_Nebrodi_S3	1580 m	S	15°	6	45%	EUNIS: R14 Perennial rocky grassland of the Italian Peninsula
1990_Nebrodi_S4	1265 m	SE	10°	6	40%	EUNIS: R14 Perennial rocky grassland of the Italian Peninsula
1990_Nebrodi_M4	1295 m	S	35°	7	30%	EUNIS: R14 Perennial rocky grassland of the Italian Peninsula
1990_Nebrodi_Su*	300 m	E	10°	10	95%	EUNIS: T211 Quercus suber forest
1990_Nebrodi_CS*	550 m	E	35-40°	10	65%	EUNIS: T211 Quercus suber forest
1990_Nebrodi_Ce*	750 m	NE	30°	10	100%	EUNIS: T19511 Southern Italic Quercus cerris forests
1990_Nebrodi_Fa*	1320 m	N	40°	11	100%	EUNIS: T1766 Northern Sicilian Fagus forests
1992_ToscoEm_ABE_rim	870 m	W	22°	6	90%	EUNIS: T3M1 Exotic spruce, fir, larch, douglas fir, deodar plantations
1992_ToscoEm_FAG_fus	946 m	N	35°	6	80%	EUNIS: T1751 Alpino-Apennine neutrophile Fagus forests
1992_ToscoEm_CAS_fru	915 m	W	31°	6	70%	EUNIS: T19C Castanea sativa forest
1992_ToscoEm_CAS_ced	925 m	W	31°	6	70%	EUNIS: T19C Castanea sativa forest
1992_ToscoEm_FAG_ced	880 m	WSW	35°	6	70%	EUNIS: T1751 Alpino-Apennine neutrophile Fagus forests
1992_ToscoEm_Incolto	930 m	W	17°	6	30%	EUNIS: R2 Mesic grasslands

**Table 2.** This table shows the frequency for each of the 177 species. The sum of the activity density (AD) is reported in the Total AD column. All species belong to the carabid family (Class: Insecta, Order: Coleoptera, Family: Carabidae).

Species	Frequency (%)	Total AD
*Calathus fuscipes* (Goeze, 1777)	55.56	94.53
*Calathus montivagus* Dejean, 1831	53.33	64.55
*Cychrus italicus* Bonelli, 1810	46.67	5.42
*Carabus lefebvrei* Dejean, 1826	44.44	9.91
*Platyderus canaliculatus* (Chaudoir, 1843)	44.44	1.87
Abax parallelepipedus subsp. parallelepipedus	42.22	97.51
*Pterostichus melas* (Creutzer, 1799)	42.22	47.03
*Notiophilus biguttatus* (Fabricius, 1779)	40.00	25.84
*Notiophilus rufipes* Curtis, 1829	33.33	20.63
*Leistus spinibarbis* (Fabricius, 1775)	33.33	0.51
*Calathus mollis* (Marsham, 1802)	31.11	7.90
*Nebria andalusia* Rambur, 1837	28.89	0.93
Nebria (Nebria) kratteri Dejean, 1831	24.44	27.50
*Calathus rotundicollis* Dejean, 1828	24.44	26.55
*Trechus obtusus* Erichson, 1837	24.44	2.14
*Leistus fulvibarbis* Dejean, 1826	24.44	0.18
*Calathus fracassii* Heyden, 1908	22.22	96.67
*Carabus convexus* Fabricius, 1775	22.22	32.20
Harpalus rufipalpis subsp. rufipalpis Sturm, 1818	22.22	1.01
*Bembidion lampros* (Herbst, 1784)	22.22	5.07
Pterostichus (Pterostichus) micans Heer, 1838	20.00	112.80
*Carabus preslii* Dejean, 1830	20.00	5.38
*Amara aenea* (DeGeer, 1774)	20.00	2.71
*Carabus variolosus* Fabricius, 1787	20.00	2.64
*Harpalus rubripes* (Duftschmid, 1812)	20.00	2.30
*Laemostenus algerinus* (Gory, 1833)	20.00	1.31
*Microlestes luctuosus* Holdhaus, 1904	20.00	0.97
*Microlestes negrita* (Wollaston, 1854)	20.00	0.53
*Nebria tibialis* (Bonelli, 1810)	17.78	1220.61
*Trichotichnus nitens* (Heer, 1837)	17.78	41.50
Harpalus decipiens subsp. decipiens Dejean, 1829	17.78	2.29
*Harpalus tardus* (Panzer, 1796)	17.78	0.38
*Laemostenus barbarus* (Lucas, 1846)	17.78	0.22
*Pterostichus unctulatus* (Duftschmid, 1812)	15.56	8.64
*Harpalus sulphuripes* Germar, 1823	15.56	1.36
*Synuchus vivalis* (Illiger, 1798)	15.56	0.28
*Carabus morbillosus* Fabricius, 1792	15.56	0.25
*Pseudomasoreus canigoulensis* (Fairmaire & Laboulbène, 1854)	15.56	0.09
*Pterostichus apenninus* (Dejean, 1831)	13.33	104.30
*Calathus rubripes* Dejean, 1831	13.33	81.56
Molops ovipennis subsp. medius Chaudoir, 1868	13.33	15.10
*Pterostichus cristatus* (L.Dufour, 1820)	13.33	7.17
*Percus passerinii* (Dejean, 1828)	13.33	6.96
*Nebria brevicollis* (Fabricius, 1792)	13.33	1.43
*Calathus melanocephalus* (Linnaeus, 1758)	13.33	0.82
*Trechus quadristriatus* (Schrank, 1781)	13.33	0.81
*Carabus planatus* Chaudoir, 1843	13.33	0.79
*Notiophilus substriatus* G.R.Waterhouse, 1833	13.33	0.26
*Harpalus attenuatus* Stephens, 1828	13.33	0.10
*Brachinus crepitans* (Linnaeus, 1758)	11.11	44.45
*Platyderus neapolitanus* (Reiche, 1855)	11.11	40.55
Pterostichus (Pterostichus) ruffoi Sciaky, 1984	11.11	1.09
*Amara sicula* Dejean, 1831	11.11	0.47
*Syntomus obscuroguttatus* (Duftschmid, 1812)	11.11	0.32
*Calosoma sycophanta* (Linnaeus, 1758)	11.11	0.11
*Harpalus serripes* (Quensel, 1806)	11.11	0.09
*Lebia cruxminor* (Linnaeus, 1758)	11.11	0.09
*Carterus dama* (P.Rossi, 1792)	11.11	0.05
*Philorhizus brandmayri* Sciaky, 1991	11.11	0.03
Pterostichus burmeisteri subsp. burmeisteri Heer, 1837	8.89	14.73
*Carabus glabratus* Paykull, 1790	8.89	8.38
*Leistus nitidus* (Duftschmid, 1812)	8.89	4.57
*Abax pilleri* Csiki, 1916	8.89	3.02
*Notiophilus pusillus* Waterhouse, 1833	8.89	2.94
*Ophonus azureus* (Fabricius, 1775)	8.89	2.22
*Trechus fairmairei* Pandellé, 1867	8.89	1.93
*Cymindis cingulata* Dejean, 1825	8.89	1.63
Carabus creutzeri subsp. baldensis Schaum, 1856	8.89	1.55
*Cicindela campestris* Linnaeus, 1758	8.89	0.96
Molops piceus subsp. tridentinus G.Müller, 1918	8.89	0.54
*Carabus rossii* Dejean, 1826	8.89	0.51
*Licinus hoffmannseggii* (Panzer, 1802)	8.89	0.26
*Tanythrix marginepunctata* (Dejean, 1831)	8.89	0.14
*Ophonus subquadratus* (Dejean, 1829)	8.89	0.11
*Amara eurynota* (Panzer, 1796)	8.89	0.06
*Licinus silphoides* (P.Rossi, 1790)	8.89	0.03
Pterostichus dubius subsp. dubius Heer, 1838	6.67	24.60
*Tanythrix edura* (Dejean, 1828)	6.67	3.66
*Pterostichus josephi* Csiki, 1930	6.67	1.72
*Carabus linnei* Panzer, 1810	6.67	1.19
*Amara nitida* Sturm, 1825	6.67	0.74
*Leistus rufomarginatus* (Duftschmid, 1812)	6.67	0.73
*Harpalus distinguendus* (Duftschmid, 1812)	6.67	0.71
Stomis (Stomis) roccai Schatzmayr, 1925	6.67	0.70
*Syntomus impressus* (Dejean, 1825)	6.67	0.58
*Microlestes levipennis* (Lucas, 1846)	6.67	0.18
*Masoreus wetterhallii* (Gyllenhal, 1813)	6.67	0.15
*Ophonus puncticeps* Stephens, 1828	6.67	0.12
*Notiophilus quadripunctatus* Dejean, 1826	6.67	0.12
*Trichotichnus laevicollis* (Duftschmid, 1812)	6.67	0.08
*Harpalus atratus* Latreille, 1804	6.67	0.03
*Leistus crenatus* Fairmaire, 1855	6.67	0.03
*Olisthopus glabricollis* (Germar, 1817)	6.67	0.03
*Bembidion lunulatum* (Geoffroy, 1785)	6.67	0.41
*Carabus violaceus* Linnaeus, 1758	4.44	6.44
Trechus (Trechus) obtusus subsp. lucanus Focarile, 1949	4.44	6.17
*Harpalus dimidiatus* (P.Rossi, 1790)	4.44	4.93
*Amara ovata* (Fabricius, 1792)	4.44	2.69
*Harpalus oblitus* Dejean, 1829	4.44	0.95
Carabus convexus subsp. dilatatus Dejean, 1826	4.44	0.90
*Poecilus cupreus* (Linnaeus, 1758)	4.44	0.68
*Pterostichus morio* (Duftschmid, 1812)	4.44	0.36
*Ophonus parallelus* (Dejean, 1829)	4.44	0.31
*Amara lucida* (Duftschmid, 1812)	4.44	0.28
*Dixus sphaerocephalus* (Olivier, 1795)	4.44	0.28
Microlestes abeillei subsp. brisouti Holdhaus, 1912	4.44	0.18
Platyderus rufus subsp. transalpinus Breit, 1914	4.44	0.13
*Paranchus albipes* (Fabricius, 1796)	4.44	0.12
Carabus violaceus subsp. picenus A. & G.B.Villa, 1839	4.44	0.10
*Calathus solieri* Bassi, 1834	4.44	0.07
*Acinopus picipes* (Olivier, 1795)	4.44	0.03
*Asaphidion rossii* (Schaum, 1857)	4.44	0.03
*Carterus rotundicollis* Rambur, 1837	4.44	0.03
*Ophonus cribricollis* (Dejean, 1829)	4.44	0.03
*Ophonus rufibarbis* (Fabricius, 1792)	4.44	0.03
*Chlaenius decipiens* (L.Dufour, 1820)	4.44	0.01
Carabus catenulatus subsp. catenulatus	4.44	0.01
*Licinus punctatulus* (Fabricius, 1792)	4.44	0.01
*Paradromius linearis* (Olivier, 1795)	4.44	0.01
*Philorhizus crucifer* (Lucas, 1846)	4.44	0.01
*Harpalus rufipes* (DeGeer, 1774)	4.44	0.42
*Pterostichus nigrita* (Paykull, 1790)	4.44	0.01
*Ophonus rupicola* (Sturm, 1818)	2.22	1.42
*Harpalus anxius* (Duftschmid, 1812)	2.22	0.56
*Anchomenus dorsalis* (Pontoppidan, 1763)	2.22	0.46
*Microlestes minutulus* (Goeze, 1777)	2.22	0.43
*Badister bullatus* (Schrank, 1798)	2.22	0.38
*Pterostichus niger* (Schaller, 1783)	2.22	0.30
*Amara similata* (Gyllenhal, 1810)	2.22	0.24
*Percus bilineatus* (Dejean, 1828)	2.22	0.13
*Acinopus baudii* A.Fiori, 1913	2.22	0.10
*Percus strictus* (Dejean, 1828)	2.22	0.10
*Bembidion rectangulum* Jacquelin du Val, 1852	2.22	0.06
*Tachys bistriatus* (Duftschmid, 1812)	2.22	0.06
*Licinus italicus* Puel, 1925	2.22	0.05
*Amara anthobia* A. & G.B.Villa, 1833	2.22	0.04
*Bembidion geniculatum* Heer, 1837	2.22	0.04
*Brachinus explodens* Duftschmid, 1812	2.22	0.04
*Cymindis axillaris* (Fabricius, 1794)	2.22	0.04
*Microlestes mauritanicus* (Lucas, 1846)	2.22	0.04
*Brachinus italicus* (Dejean, 1831)	2.22	0.03
*Clinidium canaliculatum* (O.G.Costa, 1839)	2.22	0.03
Harpalus (Harpalus) marginellus Gyllenhal, 1827	2.22	0.02
*Amara aulica* (Panzer, 1796)	2.22	0.01
*Amara equestris* (Duftschmid, 1812)	2.22	0.01
*Bembidion escherichi* Ganglbauer, 1897	2.22	0.01
*Brachinus sclopeta* (Fabricius, 1792)	2.22	0.01
*Calathus sirentensis* D'Amore Fracassi, 1908	2.22	0.01
Carabus coriaceus subsp. coriaceus	2.22	0.01
*Cychrus angustatus* Hoppe & Hornschuch, 1825	2.22	0.01
*Duvalius straneoi* Jeannel, 1931	2.22	0.01
*Laemostenus acutangulus* (L.Schaufuss, 1862)	2.22	0.01
*Lebia trimaculata* (Villers, 1789)	2.22	0.01
*Microlestes fulvibasis* (Reitter, 1901)	2.22	0.01
*Ophonus sabulicola* (Panzer, 1796)	2.22	0.01
*Philorhizus vectensis* (Rye, 1873)	2.22	0.01
Poecilus gisellae subsp. gisellae (Csiki, 1930)	2.22	0.01
*Pterostichus quadrifoveolatus* Letzner, 1852	2.22	0.01
*Zabrus orsinii* Dejean, 1831	2.22	0.01
*Acinopus megacephalus* (P.Rossi, 1794)	2.22	0.004
*Bembidion callosum* Küster, 1847	2.22	0.004
*Brachinus immaculicornis* Dejean, 1826	2.22	0.004
*Chlaenius chrysocephalus* (P.Rossi, 1790)	2.22	0.004
*Harpalus cupreus* Dejean, 1829	2.22	0.004
*Notiophilus geminatus* Dejean, 1831	2.22	0.004
*Ophonus franziniorum* Sciaky, 1987	2.22	0.003
*Asaphidion flavipes* (Linnaeus, 1760)	2.22	0.002
*Callistus lunatus* (Fabricius, 1775)	2.22	0.002
*Carabus alysidotus* Illiger, 1798	2.22	0.002
*Clivina fossor* (Linnaeus, 1758)	2.22	0.002
*Dromius agilis* (Fabricius, 1787)	2.22	0.002
*Dromius quadrimaculatus* (Linnaeus, 1758)	2.22	0.002
*Harpalus pygmaeus* Dejean, 1829	2.22	0.002
*Notiophilus palustris* (Duftschmid, 1812)	2.22	0.002
Parophonus (Parophonus) mendax (P.Rossi, 1790)	2.22	0.002
*Stomis pumicatus* (Panzer, 1796)	2.22	0.002
Stomis (Stomis) rostratus (Duftschmid, 1812)	2.22	0.001
